# Genomics Analyses of GIV and GVI Noroviruses Reveal the Distinct Clustering of Human and Animal Viruses

**DOI:** 10.3390/v11030204

**Published:** 2019-03-01

**Authors:** Lauren A. Ford-Siltz, Lisa Mullis, Yasser M. Sanad, Kentaro Tohma, Cara J. Lepore, Marli Azevedo, Gabriel I. Parra

**Affiliations:** 1 Division of Viral Products, Center for Biologics Evaluation and Research, Food and Drug Administration, Silver Spring, MD 20993, USA; lauren.siltz@fda.hhs.gov (L.A.F.-S.); kentaro.tohma@fda.hhs.gov (K.T.); caralepore@gmail.com (C.J.L.); 2 Division of Microbiology, National Center for Toxicological Research, Food and Drug Administration, Jefferson, AR 72079, USA; Lisa.Mullis@fda.hhs.gov (L.M.); marli.azevedo@fda.hhs.gov (M.A.); 3 Department of Agriculture, School of Agriculture, Fisheries, and Human Sciences, University of Arkansas, Pine Bluff, AR 71601, USA; sanad.1@osu.edu; 4 Department of Parasitology and Animal Diseases, Veterinary Research Division, National Research Centre, Giza 12622, Egypt

**Keywords:** norovirus, canine norovirus, inter-species transmission

## Abstract

Noroviruses are highly diverse viruses that are the major viral cause of acute gastroenteritis in humans. Although these viruses can infect multiple mammalian species, their potential for zoonosis is not well understood, especially within Genogroup IV (GIV), which contains viruses that infect humans, canines, and felines. The study of GIV viruses has been, in part, hindered by the limited number of complete genomes. Here, we developed a full-genome amplicon-based platform that facilitated the sequencing of canine noroviruses circulating in the United States. Eight novel nearly full-length canine norovirus genomes and two nearly complete VP1 sequences, including four GIV.2, three GVI.1, and three GVI.2 viruses, were successfully obtained. Only animal strains exhibited GVI/GIV chimeric viruses, demonstrating restrictions in norovirus recombination. Using genomic, phylogenetic, and structural analyses, we show that differences within the major capsid protein and the non-structural proteins of GIV and GVI noroviruses could potentially limit cross-species transmission between humans, canines, and felines.

## 1. Introduction

Noroviruses are a highly diverse group of viruses from the *Caliciviridae* family. They have a positive-sense RNA genome of approximately 7.5 kilobases that is organized into at least three open reading frames (ORFs). ORF1 encodes a polyprotein that is co-translationally cleaved into the individual non-structural (NS) proteins involved in replication: NS1/2 (N-term), NS3 (NTPase), NS4 (3A-like), NS5 (VPg), NS6 (Protease), and NS7 (Polymerase). ORF2 encodes the major capsid protein, VP1, which forms the virus capsid. The norovirus VP1 protein is divided into two structural domains: (i) the shell domain, the conserved region that makes up the inner core of the capsid; and (ii) the protruding domain, a highly variable region containing protective epitopes and motifs that interact with attachment factors, such as histo-blood group antigens (HBGAs), that could facilitate viral infection [1,2,3,4]. ORF3 encodes the minor capsid protein, VP2, which is thought to play a role in capsid stabilization and viral entry [5,6,7]. Both VP1 and VP2 are expressed from a subgenomic RNA that is synthesized during replication and includes the ORF2, ORF3, 3′-untranslated region (3′-UTR), and the terminal poly-A tail [8].

Noroviruses are classified into at least seven genogroups based on the genetic diversity of VP1; genogroups I, II, and IV (or GI, GII, and GIV) include viruses which infect humans. Noroviruses have also been identified in cows and sheep (GIII), mice and rats (GV), pigs (GII), canines (GIV, GVI, GVII), and felines (GIV, GVI), suggesting a strong segregation of genogroups based on taxonomic orders. Among humans, they are the most common viral cause of acute gastroenteritis, with symptoms including diarrhea, vomiting, fever, headache, and myalgia. Although most infections are mild with symptoms lasting 12–48 h, noroviruses can produce severe disease in high-risk populations, such as immunocompromised or malnourished individuals and the elderly. Thus, they have been implicated in an estimated 200,000 deaths per year, mainly in developing countries [9].

In contrast to human noroviruses, the importance of noroviruses as pathogens of canines and felines has not been extensively analyzed. Studies have shown that canine norovirus can be detected in varying percentages (~2–40%) in dog fecal samples [10,11,12], and the virus was more likely to be detected in dogs experiencing symptoms of gastroenteritis [12]. Likewise, feline norovirus was suggested to be responsible for an outbreak of gastroenteritis in kittens from an animal shelter in the United States [13]. Moreover, norovirus-specific antibodies have been detected in cats and dogs [14,15,16]. Thus, while animal noroviruses from GIV and GVI are widespread, they seem to have a moderate impact on disease in canines and felines. Although GVI viruses have only been detected in animals, the similarity of human, canine, and feline noroviruses within GIV raises concerns over the potential for zoonosis. Several studies have suggested that dogs may play a role in the transmission of human noroviruses and that humans present antibodies against canine norovirus [17,18,19,20]. However, no study has been able to present concrete evidence that human norovirus causes disease and/or replicates within animals or vice versa.

Despite being discovered over 20 years ago and that similar viruses have been identified in humans and companion animals, there are a limited number of complete genome sequences available for GIV and GVI noroviruses. The goal for this study was to increase the number of complete genome sequences from canine noroviruses to gain insights into their diversity and potential for inter-species transmission. We report newly designed primers that were used to amplify and facilitate the sequencing of viral genomes from GIV and GVI noroviruses detected in samples from domestic dogs in the United States. Sequence, phylogenetic, and structural-modeling analyses revealed that GIV and GVI strains are separated into animal and human lineages with multiple amino acid (aa) differences that could potentially hinder inter-species transmission.

## 2. Materials and Methods

### 2.1. Sample Collection

Stool samples or rectal swabs were collected from domestic canines that had been admitted to veterinary care for symptoms of gastroenteritis in Arkansas, USA. Collections from Cohort 1 spanned from 2009–2013 and collections from Cohort 2 were from 2017. Samples were suspended in MEM (4% for Cohort 1 and 10% for Cohort 2) and stored at −80 °C.

### 2.2. RNA Extraction and RT-PCR

RNA was extracted from 115 μL of suspension with the MagMax Viral RNA Isolation Kit (ThermoFisher Scientific, Sunnyvale, CA, USA). cDNA was synthesized using the Tx30SXN primer (5′-GACTAGTTCTAGATCGCGAGCGGCCGCCCTTTTTTTTTTTTTTTTTTTTTTTTTTTTTTT-3′) [21] at a final concentration of 10 µM with the Maxima First Strand cDNA Synthesis Kit (ThermoFisher Scientific, Sunnyvale, CA, USA), according to the manufacturer’s protocol, except that 0.1 µL of enzyme was added per reaction. Samples were screened for the presence of norovirus with primers that amplify a short region of the polymerase, JV12Y (5′-ATACCACTATGATGCAGAYTA-3′) and JV13I (5′-TCATCATCACCATAGAAIGAG-3′). Samples positive for norovirus genomes were screened with new primers that were designed based on previously determined GIV and GVI sequences to amplify the subgenomic (SG) region and/or the full-length (FL) genome: Canine_1-23_For (5′-GTGAATGATGATGGCGTCTAACG-3′) and Tx30SXN amplifies the subgenomic region, while Canine_1-34_For (5′-GTGAATGATGATGGCGTCTAACGACGCTATCCCC-3′) and Tx30SXN amplifies the full-length genome and the subgenomic region. The protocol for full-genome amplification, which was also used for subgenomic amplification, is described elsewhere [22]. RT-PCR products were run on 1% agarose gels, and bands corresponding to the full-length genome (~7800 bp) and the subgenomic region (~2500 bp) were excised from the gels and purified using the QIAquick Gel Extraction Kit (Qiagen, Hilden, Germany). Samples were sequenced using the Illumina MiSeq. The 3′ ends were amplified with a semi-nested PCR using the gel-purified FL or SG amplicons as templates. The primers used for PCR were Canine_VP2_For1 (5′-GTTGACTGGAATGGCAC-3′) and Tx30SXN. The 3′ ends were sequenced with Sanger sequencing using the Canine_VP2_For1 primer.

### 2.3. Sequence and Phylogenetic Analyses

Sequence assembly was performed with the High-performance Integrated Virtual Environment (HIVE) platform using the HIVE-hexagon reference guided alignment tool [23,24], which is an algorithm designed to align next-generation sequencing (NGS) reads to reference genomes. Sequence alignments and phylogenetic analyses were performed with the MEGA software (version 7.0.18) [25] or with MAFFT (version 7) [26], depending on the complexity of the alignment. Trees were modified in FigTree (version 1.4.3). The accession numbers for previously published sequences (as of April 2018) used in the analyses, as well as for the new full-length canine genomes and subgenomic amplicons, are listed in Table S1. The Shannon entropy, a measure of the variation of a protein sequence alignment, was determined using the Shannon Entropy-One tool using amino acid class equivalents for the calculation (available at https://www.hiv.lanl.gov/content/sequence/ENTROPY/entropy_one.html). Entropy values for each position were plotted in GraphPad Prism v7 (San Diego, CA, USA).

### 2.4. Structural Modeling

A model of the P-domain dimer of canine AN843 based on the crystal structure of a feline norovirus P-domain (4QUZ) was inferred using the Iterative Threading Assembly Refinement (I-TASSER) server (https://zhanglab.ccmb.med.umich.edu/). Protein models were visualized and analyzed with UCSF Chimera (version 1.11), developed by the Resource for Biocomputing, Visualization, and Informatics at the University of California, San Francisco, USA [27].

## 3. Results

### 3.1. Amplification, Sequencing, and Characterization of Novel Canine Norovirus Strains

**Figure 1:**
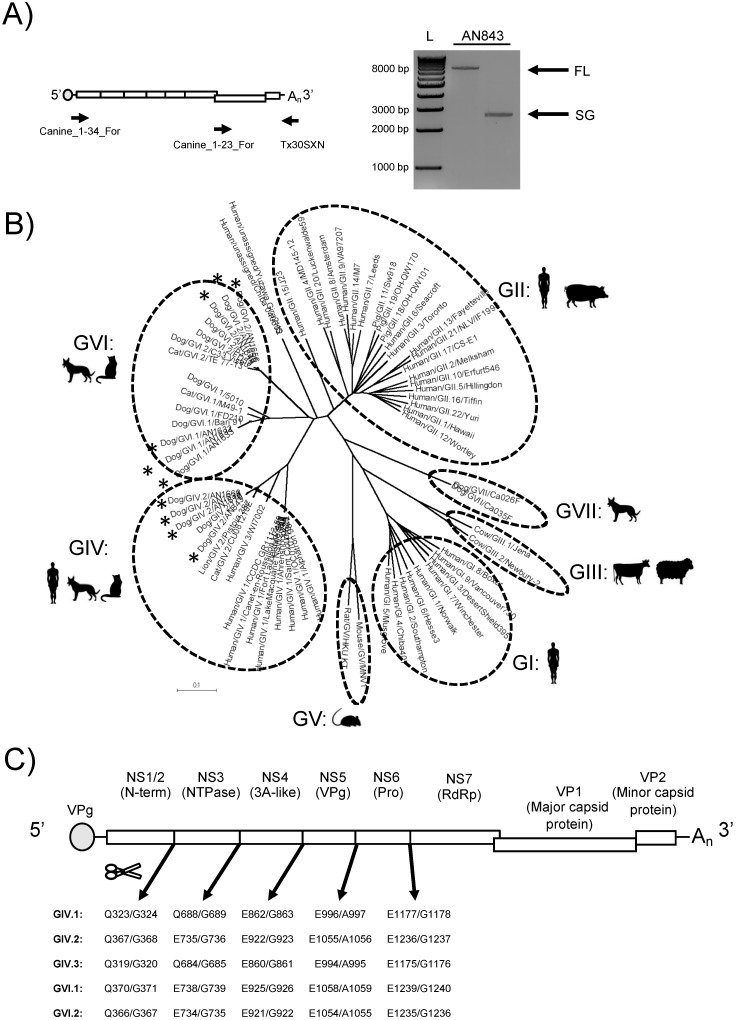
Amplification and characterization of novel canine norovirus strains. (**A**) Scheme used to amplify nearly full-length (FL) genome and the subgenomic (SG) region (left). The primers used for amplification are represented by arrows below the genome. Electrophoresis gels showing amplicons obtained from FL and SG RT-PCR from a representative sample (right). L = 1 kb ladder. (**B**) Phylogenetic analyses of norovirus major capsid protein (VP1) from representative strains from each genogroup. Phylogenetic tree of the complete aa sequence of VP1 based on the Neighbor Joining method. The phylogenetic tree was calculated with MEGA software (version 7.0.18). Strains described in this study are labeled with an asterisk (*). (**C**) Genome map of canine norovirus with predicted ORF1 cleavage sites for genotypes within GIV and GVI. The 5′ end of the genome is predicted to be capped with the VPg protein (encoded by NS5), while the 3′ end consists of a short 3′ untranslated region (3′-UTR) and a poly-A tail. AA numbers of the GVI noroviruses are based on the feline norovirus strain CU081210E (JF781268).

Rectal swabs and stool samples were collected from two cohorts of canines admitted to veterinary care for symptoms of gastroenteritis in Arkansas, USA. The presence of norovirus genomes was confirmed in 34/89 (38.2%) samples in Cohort 1 (2009–2013) and 12/124 (9.7%) samples in Cohort 2 (2017) by reverse-transcription (RT)-PCR targeting the polymerase region [12,28]. Samples positive for noroviruses were tested for full-genome sequencing by using primers that anneal to the conserved 5′ end and the 3′ poly-A tail of the genomes (Figure 1A). We successfully amplified eight canine norovirus nearly complete genomes, one from Cohort 1 and seven from Cohort 2. Two additional canine norovirus subgenomic amplicons were isolated from Cohort 2 using primers that preferentially amplify the subgenomic region (Figure 1A). The nearly full-length and subgenomic amplicons were purified and sequenced using next-generation sequencing (NGS) technologies with a depth of coverage that ranged between 5300 and 21,000 reads per site.

**Figure 2:**
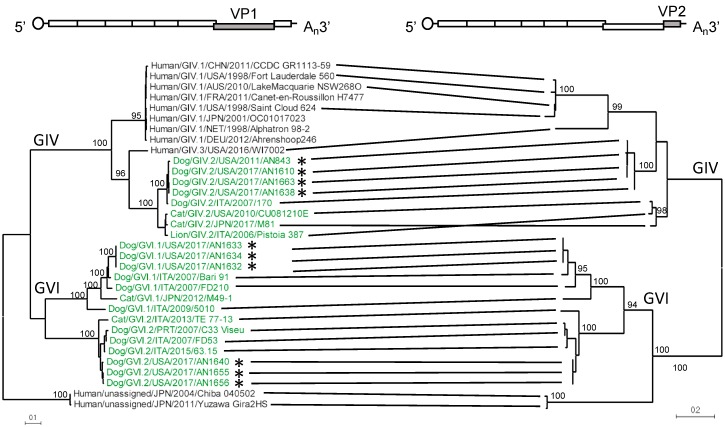
Phylogenetic analyses of GIV and GVI norovirus major (VP1) and minor (VP2) capsid proteins reveal clustering into distinct lineages and suggest their co-evolution. Maximum likelihood tree of the complete aa sequence of VP1 based on the Jones-Taylor-Thornton (JTT) matrix-based model. The phylogenetic tree was calculated with MEGA software (version 7.0.18). Strains described in this study are labeled with an asterisk (*). Strains highlighted in green and black represent animal and human noroviruses, respectively. Co-evolution is indicated by connecting strains from each of the phylogenetic trees. A diagram of the genome indicating the region used for the analyses (light grey) is shown on top.

To characterize the sequenced noroviruses, we mined the public databases for complete norovirus VP1 sequences, representing viruses from all genogroups (GI–GVII). Sequence and phylogenetic analyses showed that the novel strains were determined to be GIV.2 (four strains), GVI.1 (three strains), and GVI.2 (three strains) (Figure 1B). We successfully amplified full-length and/or the subgenomic amplicon from 1/34 (2.9%) of norovirus-positive samples from Cohort 1 (2009–2013) and from 9/12 (75%) of norovirus-positive samples from Cohort 2 (2017). The large discrepancy in successful genome amplification between these two cohorts could be explained by the age of the samples, the large diversity of canine noroviruses, or the differences in dilution of the original suspensions (4% vs. 10%). The new GIV.2 strains cluster together with canine strain 170, which was detected in Italy in 2007. The two feline strains, Pistoia 387 (lion) and CU081210E (domestic cat), form a branch separate from the canine strains. Likewise, the novel canine GVI.1 and GVI.2 strains clustered apart from the sole feline GVI.1 and GVI.2 strains, respectively (Figure 1B and Figure 2). We also looked at the GIV and GVI aa diversity within and between genotypes of the complete VP1 protein. As expected, there were a greater number of aa differences between genotypes than within a genotype (202.1 vs. 16.11 differences, *p* < 0.0001) (Figure S1).

The predicted cleavage sites for the NS proteins from each genotype within GIV and GVI were identified based on the high conservation of the cleavage sites between norovirus genogroups (Figure 1C). ORF2, which encodes for VP1, is predicted to start between nucleotide (nt) positions 5200 and 5225 and to be between 1716 and 1749 nts (572–583 aa) in length. ORF3, which encodes for the minor structural protein VP2, is estimated to start between nt positions 6959 and 6961 and to be between 765 and 840 nts (255–280 aa) in length. The 3′ end of the genome is flanked by a 3′-UTR that ranged from 111 to 136 nts and a poly-A tail. While the hotspot for recombination has been mapped at the junction of ORF1/ORF2 [29], studies in human and murine noroviruses have found recombination events at the ORF2/ORF3 junction [30,31]. To determine the evolutionary relationship between the major and minor capsid proteins of GIV and GVI noroviruses, we conducted phylogenetic analysis using complete VP2 sequences of GIV and GVI noroviruses and showed that the strains clustered in a genotype-dependent manner, similar to the clustering of VP1 (Figure 2).

### 3.2. Structural Analyses of VP1 Reveal Species-Specific Differences between Human, Canine, and Feline Capsid Proteins

**Figure 3:**
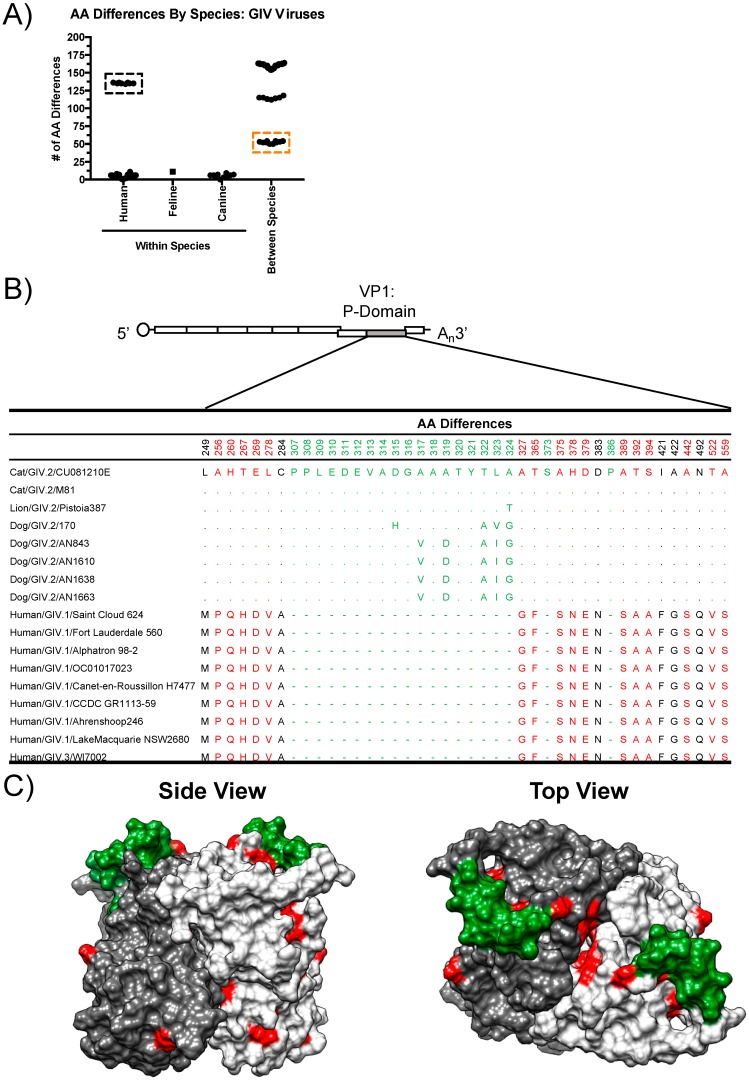
Human and animal GIV noroviruses present major differences in their VP1 sequences. (**A**) Number of amino acid differences within and between species of GIV viruses. A black box denotes aa differences between GIV.1 and GIV.3 human noroviruses. An orange box highlights aa differences of feline versus canine norovirus. (**B**) Table of conserved mutations within the P-domain of human and animal GIV capsid proteins. Surface-exposed mutations are highlighted in red and insertions are highlighted in green. A diagram of the genome indicating the region used for the analyses (light grey) is shown on top. (**C**) Model of the P-domain dimer of AN843 (GIV.2). The model is based on the crystal structure of GIV.2 feline norovirus CU081210E (PDB ID = 4QUZ) and consists of aa residues 225 to 565. The P-domain monomers are colored in light and dark grey. Surface-exposed aa differences between the human and animal structures are highlighted in red and insertions are highlighted in green. The model, which was produced with the I-TASSER server, was visualized in Chimera (version 1.11).

To define the differences between human, canine, and feline noroviruses, we analyzed the number of aa differences within the VP1 of GIV noroviruses by species. The number of aa differences within each species was lower than between species (<12 aa differences within each species compared to 50–164 aa differences between species), even when comparing canine norovirus to feline norovirus, which are both within the GIV.2 genotype (Figure 3A, orange box). This variation helps to explain the separation of canine and feline noroviruses in the phylogenetic tree (Figure 2). Of note, the GIV.3 human norovirus appears to be remarkably different from the GIV.1 human strains (Figure 3A, black box).

To determine whether order-specific aa differences appear on the surface of VP1, we aligned the GIV.2 VP1 aa sequences and identified all conserved residues that were different between human and animal viruses (Figure 3B). Interestingly, the GIV noroviruses contain many differences within the VP1 protein that differentiate GIV.1, GIV.2, and GIV.3 viruses (Table S2), suggesting that these particular residues likely help to define the GIV genotypes. To determine the location of the differences, the I-TASSER server was used to predict a model of the canine norovirus AN843 P-domain dimer based on the crystal structure of feline norovirus CU081210E (Protein Data Base [PDB] ID = 4QUZ) [13,32]. Although aa variation exists between strains, for this analysis, only aa residues that were conserved at the human or animal level were considered. Sixteen out of 22 (72.7%) of the residues were located on the surface of the structure. Notably, the GIV.2 P-domain contains a large insertion that forms a loop on the top of the structure (residues 307–324) (Figure 3C). This insertion was previously identified in a feline norovirus strain [32]. All canine and feline noroviruses within GIV.2 and GVI share this insertion near the top of the structure, in contrast to the human GIV.1 and GIV.3 noroviruses (Figure 3B, Table S3). We also aligned all GIV.2 VP1 sequences to compare aa differences between canine and feline viruses (Figure S2A). Twenty-one out of 26 (80.8%) of the residues were located on the surface of the P-domain (Figure S2B). Taken together, the VP1 proteins of canine and feline norovirus contain multiple surface-exposed differences and a large insertion that differentiates them from the human norovirus VP1 protein.

### 3.3. Analyses of Non-Structural Proteins Suggest Order-Specific Differences within the Replication Machinery

**Figure 4:**
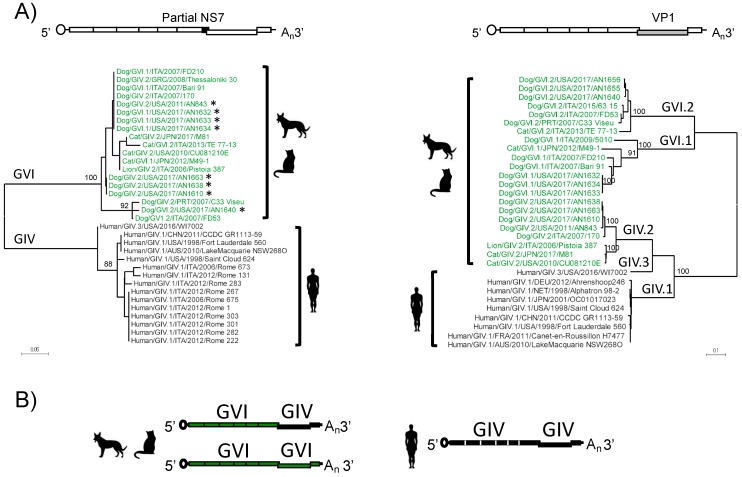
The GIV and GVI polymerases are clustered into two distinct groups. (**A**) A maximum likelihood tree of the partial polymerase (aa 427–512) based on the JTT matrix-based model. The phylogenetic tree was calculated with MEGA software (version 7.0.18). Strains described in this study are labeled with an asterisk (*). Strains highlighted in green and black represent animal and human noroviruses, respectively. A diagram of the genome indicating the region used for the analyses (black) is shown on top. (**B**) An illustration of recombinant norovirus genome organization representing the GIV and GVI viruses.

The addition of eight new canine norovirus nearly full-length genomes allows for better analyses of the NS proteins involved in replication and host interactions. Conventionally, norovirus classification has been based on both the polymerase and VP1 genotypes [8]. The capsid genotyping is based on the complete ORF2 sequence; however, polymerase typing is based on sequences from the C-terminal region of the polymerase (NS7). Thus, we first constructed a phylogenetic tree using the C-terminal region of the polymerase, as there are more sequences of this region available in the database. Interestingly, phylogenetic analysis of this region (aa 427–512) revealed two distinct groups: one (GIV) included only human strains, and another (GVI) included all animal strains (Figure 4). Notably, clustering into these groups was independent of the capsid genotype, as in animals, the GVI polymerases were associated with both GIV.2 and GVI capsids, while the human GIV polymerases were only associated with GIV capsids. Thus, genogroup recombination appears to be relaxed for the animal noroviruses.

**Figure 5:**
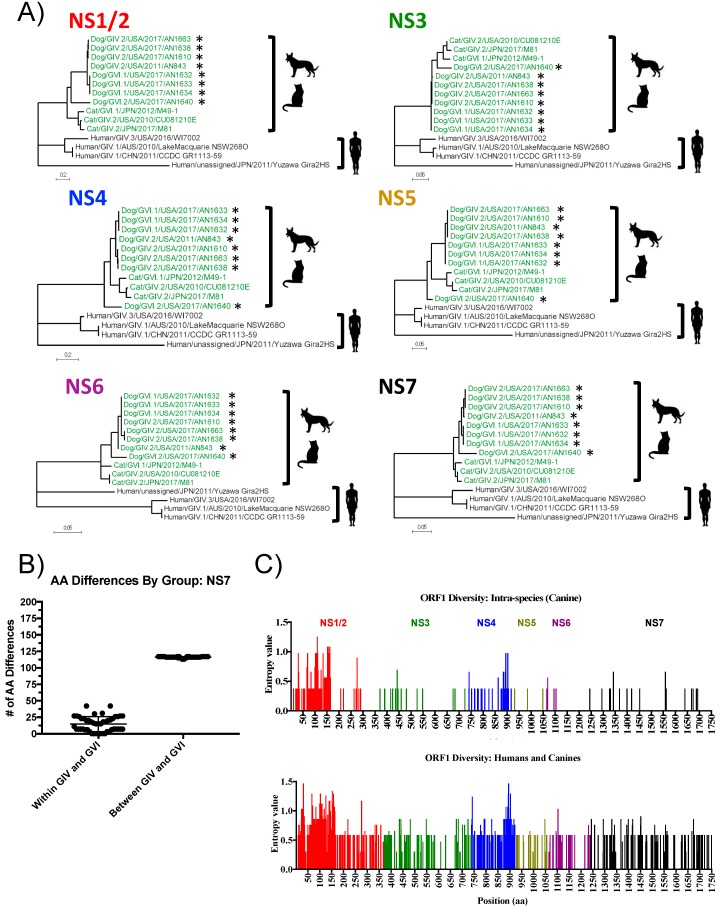
Analyses of GIV and GVI norovirus ORF1 reveal high diversity within regions of the non-structural proteins and the clustering into two distinct groups. (**A**) Maximum likelihood trees of the complete aa sequence of the individual non-structural proteins based on the Le Gascuel (2008) model. The phylogenetic trees were calculated with MEGA software (version 7.0.18). Strains described in this study are labeled with an asterisk (*). Strains highlighted in green and black represent animal and human noroviruses, respectively. (**B**) AA differences of the complete polymerase within and between groups (GIV and GVI). (**C**) Diversity plots, as calculated by Shannon entropy, spanning the ORF1 of canine noroviruses (intra-species, top panel) and canine and human noroviruses (inter-species, bottom panel). The values for each individual NS protein are differentiated by color.

To further elucidate evolutionary differences within the NS proteins that may discriminate human, canine, and feline noroviruses, we constructed phylogenetic trees of the individual NS proteins using the available full-length norovirus sequences. In agreement with analyses done with the partial polymerase-encoding region (Figure 4), phylogenetic analysis of the complete polymerase also revealed the clustering into the GIV (human) and GVI (animal) groups (Figure 5A). Similar clustering was seen with the other individual NS proteins (NS1/2, NS3, NS4, NS5, and NS6) (Figure 5A), suggesting co-evolution of the proteins within ORF1 with a marked host specificity. Analysis of the sequence diversity of GIV and GVI norovirus polymerases revealed high conservation within each group and large numbers of aa differences between groups (Figure 5B, Table S4). The human norovirus polymerase proteins (GIV) were ~22.7% different compared to those of canines and felines (GVI); in contrast, the difference between canine and feline polymerases (4.0%) fell within the range of aa differences within each species (1.9 to 5.5%). Sixty-four conserved residues (12.5% of the total aa residues) that distinguish human from animal polymerases were identified; these residues were highlighted in the model of the canine polymerase (Figure S3). Most of the differences were located on the surface of the polymerase, where they could potentially interact with host and viral proteins involved in the formation of the replication complex.

To determine the sequence differences within the ORF1 polyprotein, we calculated the Shannon entropy value, or the measure of uncertainty at each nucleotide or amino acid position, of the complete aa sequence alignment of the GIV and GVI canine noroviruses (intraspecies). The entropy values across ORF1 were low (mean: 0.044, range: 0–0.13), suggesting that the canine NS proteins are highly conserved. Most variation was observed within NS1/2, particularly at the N-terminal end, and within NS4 (Figure 5C, top panel). The large entropy values detected at the NS1/2 region were mostly due to aa deletions within the GVI.1 genotype, and the high divergence of the GVI.2 sequence (AN1640) from the other canine sequences. The entropy values of a canine and feline norovirus alignment were similar (Figure S2C). However, when human and canine viruses were considered, the Shannon entropy values spanning the whole ORF1 region were high (mean: 0.17, range: 0–1.47, Figure 5C, bottom panel), reinforcing the differences between human and animal noroviruses.

## 4. Discussion

Noroviruses present an extreme genetic diversity that is, in part, associated with the large number of different mammals that the viruses infect. Despite this large host range, each of the virus genogroups seems to be restricted to specific taxonomic groups. Numerous attempts have been made to develop animal models to study human noroviruses, with varying success [33,34,35,36,37,38,39], but this strong segregation at the host level could be involved in limiting the robustness of most of these models. Recently, an animal model, which recapitulated the natural course of infection seen in humans, has been reported with feline noroviruses [40]. Noroviruses infecting felines have been classified as GIV and GVI, with GIV viruses also being detected in humans. While extensive genetic information is available for noroviruses infecting humans [22], little is known about canine and feline noroviruses, which complicates studies on their evolution and biological properties. Simplification of virus genome amplification from clinical samples has greatly advanced genomics analyses for different human viruses [41,42,43]. We designed primers that allowed broad amplification of the full-length genome of GIV and GVI noroviruses and successfully amplified and sequenced full-length and/or subgenomic regions of noroviruses detected in dogs. The new data facilitate genomic studies for GIV and GVI noroviruses, which present evidence that differences in both structural and non-structural proteins segregate human and animal noroviruses into distinct genetic groups.

Attachment and entry is an important first step of the viral life cycle and accounts for one of the first barriers to cross-species transmission and infection. In noroviruses, it was recently shown that the expression of murine norovirus receptor (molecules from the CD300 family) allows murine norovirus (GV.1) replication in non-murine mammalian cells that were otherwise resistant to the infection [44,45]. Here, we show that the major capsid protein, VP1, is conserved among samples from the same taxonomic order, but highly variable between orders, specifically between humans and carnivores. Although the cellular receptor and the exact motifs involved in attachment to HBGA carbohydrates have not been identified for feline or canine noroviruses [32], both GIV and GVI viruses have been shown to interact with HBGA carbohydrates, specifically the H and A antigens [18]. A major difference between human and animal noroviruses is a large insertion that maps at between beta sheets β2 and β3 on the top of the P domain [32,46], thus potentially restricting the interaction of VP1 and host factors (carbohydrates and/or receptor) required for viral entry. 

While the precise role of norovirus VP2 has not been conclusively demonstrated, previous studies have shown that VP2 could help virion stability or could play a role in regulating the maturation of antigen-presenting cells and of protective immunity in a virus strain-specific manner [47,48,49]. Moreover, by examining the cryo-electron microscopy structures of feline calicivirus, a *Vesivirus*, it was shown that VP2 forms a portal-like assembly that creates a pore in the capsid shell that could function as a channel for the delivery of the viral genome into the host cell cytoplasm [7]. In this pore, VP2 interacts with the protruding domains of the capsid. Strain-specific VP1–VP2 interactions have been shown for GII.4 noroviruses, resulting in the covariation of the P domain of VP1 and the central region of VP2 [50]. In our genomic analyses, VP2 clustered into groups similar to that of VP1, further supporting that co-evolution between these two proteins is functionally driven. Norovirus VP2 is over two times larger in length as compared to that of Vesiviruses [6,51], thus it remains to be determined whether the function of norovirus VP2 is similar to that of the feline calicivirus.

The addition of eight canine norovirus genomes allows for sequence analyses of the canine GIV and GVI NS proteins for the first time. Phylogenetic analyses of the individual NS proteins revealed the clustering into two distinct groups: one comprised all animal GIV and GVI noroviruses, while the other comprised the human GIV viruses. Thus, animal noroviruses presented GVI polymerases with GIV.2, GVI.1, and GVI.2 capsids, while humans noroviruses presented GIV polymerases with GIV.1 and GIV.3 capsids. These data support inter-genogroup recombination events in noroviruses, something considered rare for this group of viruses [10,52,53]. Moreover, these analyses also report inter-genogroup recombinant norovirus strains in canines from the United States, expanding their geographic distribution at the global scale. Minor differences between the animal noroviruses, which can be putatively divided into two lineages, can also lead to intra-group recombination at the ORF1/ORF2 junction between lineages [11]. Together, this suggests that both structural and non-structural proteins differentiate human and animal noroviruses.

Several studies have suggested that NS proteins of other viruses can be involved in determining host range and can contribute to pathogenicity [54,55,56,57]. The effects of the NS proteins range from mediating innate immunity [54] to influencing the compatibility of proteins from the viral replicative machinery with host proteins [55,56,57]. Sequence analyses of the GIV and GVI norovirus genomes revealed high diversity throughout the ORF1 region, particularly when comparing human and animal strains. Although the functions of some of the norovirus NS proteins are not completely elucidated, current data suggest that these proteins are involved in the formation of the viral replication complex through interactions with host proteins [58,59,60,61,62,63,64]. Comparison of the crystal structures of human and animal norovirus polymerases revealed differences on the surface and within the polymerase. Whether these differences have any effect on mediating binding to species-specific host proteins involved in the viral life cycle, as shown for other viruses with zoonotic potential [57], remains to be determined. 

In conclusion, we present a simple method to sequence the nearly full-length genome of GIV and GVI noroviruses that could facilitate closing the knowledge gap for genetic information of noroviruses circulating in canines and felines. Using novel genomes sequenced with this method, we showed that (i) inter-genogroup GIV/GVI recombinants circulate in canines and felines from different continents, and (ii) human and animal GIV and GVI noroviruses clustered separately at non-structural and structural proteins, which may restrict inter-species transmission. The development of robust cell culture systems and animal models for human and animal noroviruses would more definitively support or dispute the claim of inter-species transmission [17,18,19].

## Supplementary Materials

The following are available online at https://www.mdpi.com/1999-4915/11/3/204/s1, Figure S1: Number of AA differences within and between genotypes of the complete VP1 of GIV and GVI noroviruses, Figure S2: Structural modeling reveals aa differences within the VP1 of canine and feline noroviruses, Figure S3: The GIV and GVI polymerases contain distinct aa differences, Table S1: List of human, canine, and feline noroviruses used for analyses, Table S2: Amino acid differences within VP1 that differentiate the genotype of GIV noroviruses, Table S3: Amino acid alignment within VP1 (aa 301–337) of GIV and GVI noroviruses, Table S4: Number of amino acid differences (% differences) of the complete polymerase (total of 511 residues) within and between species.
