# Supplementary material for: Genomics Analyses of GIV and GVI Noroviruses Reveal the Distinct Clustering of Human and Animal Viruses
Source: Viruses. 2019 Mar 1;11(3):204. doi: 10.3390/v11030204 (PMC6466045; doi:10.3390/v11030204)
Supplement: Supplementary file 1 [file viruses-11-00204-s001.zip › Figure S1.pdf]

Fig. S1

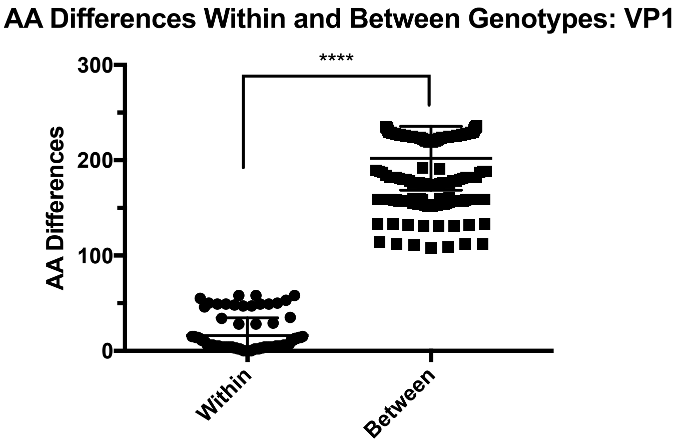

Supplemental Figure 1. Number of AA differences within and between genotypes of the complete VP1 of GIV and GVI noroviruses. **** represents p<0.0001.
