# Supplementary material for: Genomics Analyses of GIV and GVI Noroviruses Reveal the Distinct Clustering of Human and Animal Viruses
Source: Viruses. 2019 Mar 1;11(3):204. doi: 10.3390/v11030204 (PMC6466045; doi:10.3390/v11030204)
Supplement: Supplementary file 1 [file viruses-11-00204-s001.zip › Figure S2.pdf]

Fig. S2

A)

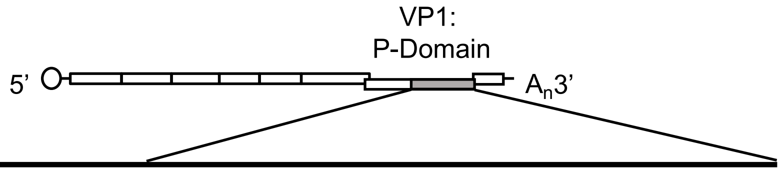

AA Differences in P-Domain

	233	236	280	289	293	298	303	322	333	339	341	348	363	385	412	428	439	449	457	477	479	492	494	497	543	557	567	575	577
Dog/GIV.2/AN843	T	E	V	T	H	E	S	A	I	N	Q	E	I	R	E	N	E	N	K	S	S	D	V	F	D	N	N	A	N
Dog/GIV.2/AN1610	.	.	.	.	.	.	.	.	.	.	.	.	.	.	.	.	.	.	.	.	.	.	.	.	.	.	.	.	.
Dog/GIV.2/AN1638	.	.	.	.	.	.	.	.	.	.	.	.	.	.	.	.	.	.	.	.	.	.	.	.	.	.	.	.	.
Dog/GIV.2/AN1663	.	.	.	.	.	.	.	.	.	.	.	.	.	.	.	.	.	.	.	.	.	.	.	.	.	.	.	.	.
Dog/GIV.2/170	.	.	.	.	.	.	.	.	.	.	.	.	.	.	.	.	.	.	.	.	.	.	.	.	.	.	.	.	.
Cat/GIV.2/CU081210E	V	D	I	L	Q	G	T	T	L	D	R	D	V	T	Q	S	D	D	R	A	A	E	I	L	N	S	S	S	A
Lion/GIV.2/Pistoia387	V	D	I	L	Q	G	T	T	L	D	R	D	V	T	Q	S	D	D	R	A	A	E	I	L	N	S	S	S	A

B)

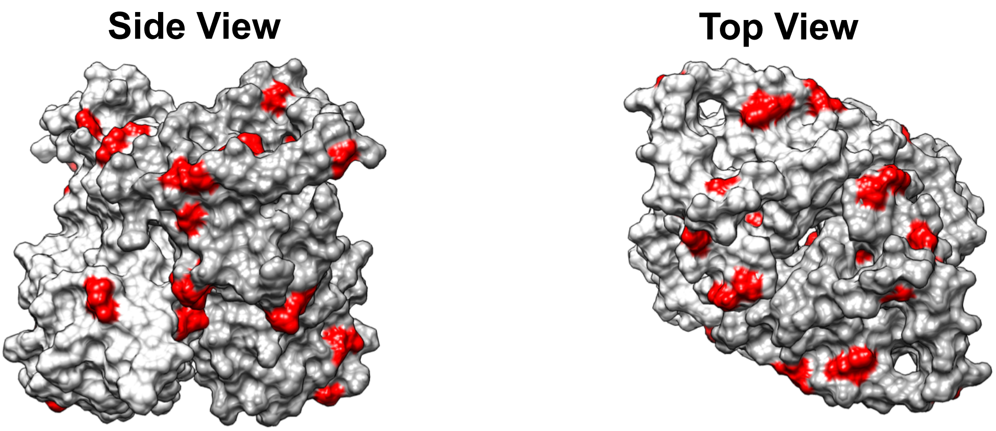

C)

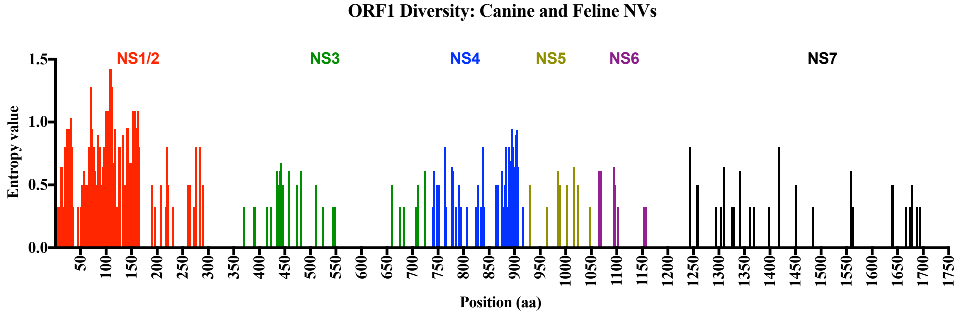

Supplemental Figure 2. Structural modeling reveals aa differences within the VP1 of canine and feline noroviruses. (A) Amino acid variation between canine and feline norovirus P-domains. Amino acid numbers are based on the complete GIV.2 VP1 alignment. Surface-exposed residues are noted in red and correspond to the highlighted residues in Figure 3A. Small dots indicate conserved residues compared to the initial sequence (AN843). A diagram of the genome indicating the region used for the analyses (light grey) is shown on top. (B) Model of the P-domain dimer of AN843 (GIV.2). The model is based on the crystal structure of GIV.2 feline norovirus CU081210E (PDB ID = 4QUZ) and consists of aa residues 225 to 565. The P-domain monomers are colored in light and dark grey. Surface-exposed aa differences between the canine and feline structures are highlighted in red. The model, which was produced with the I-TASSER server, was visualized in Chimera (version 1.11). (C) Diversity plots, as calculated by Shannon entropy, spanning the ORF1 of canine and feline norovirus. The values for each individual NS protein are differentiated by color.
