# Supplementary material for: Genomics Analyses of GIV and GVI Noroviruses Reveal the Distinct Clustering of Human and Animal Viruses
Source: Viruses. 2019 Mar 1;11(3):204. doi: 10.3390/v11030204 (PMC6466045; doi:10.3390/v11030204)
Supplement: Supplementary file 1 [file viruses-11-00204-s001.zip › Figure S3.pdf]

Fig. S3

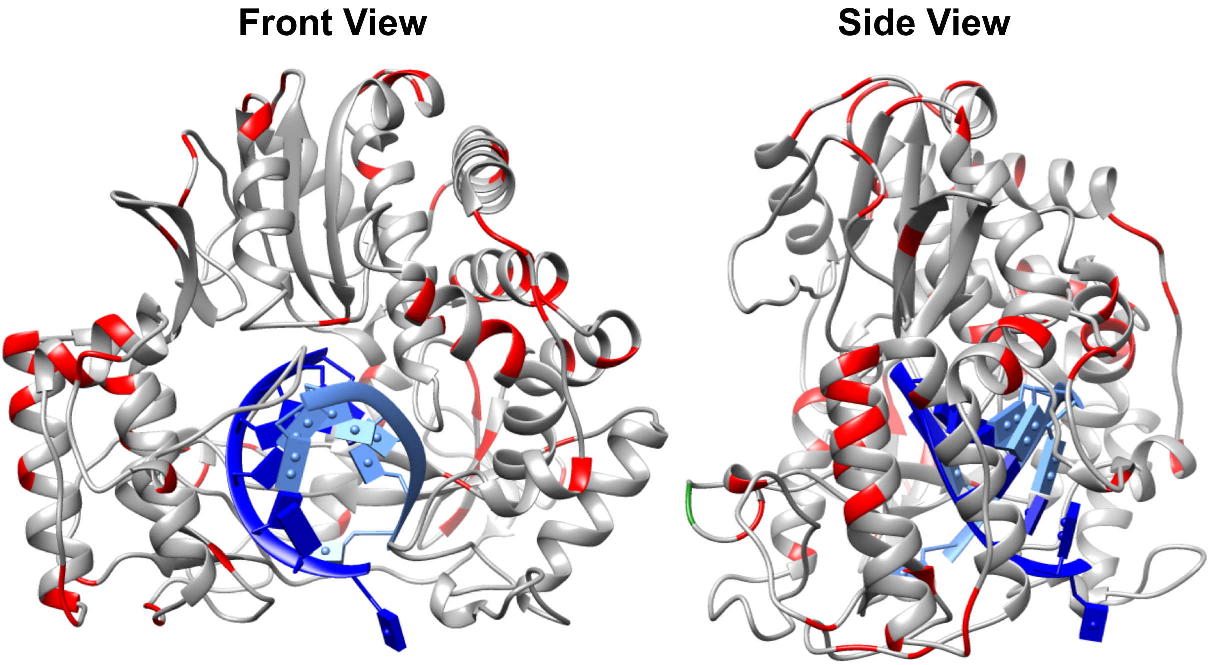

Supplemental Figure 3. The GIV and GVI polymerases contain distinct aa differences. A model of the polymerase of AN843 (GIV.2) surrounding a newly-transcribed RNA molecule (in blue). The model is based on the crystal structure of a GII human norovirus (PDB ID = 4QPX). Conserved aa differences between the GIV human polymerase structure to all canine and feline polymerases are highlighted in red. An insertion within the animal polymerases is highlighted in green. The model, which was produced with the I-TASSER server, was visualized in Chimera (version 1.11).
