# Supplementary material for: Genomics Analyses of GIV and GVI Noroviruses Reveal the Distinct Clustering of Human and Animal Viruses
Source: Viruses. 2019 Mar 1;11(3):204. doi: 10.3390/v11030204 (PMC6466045; doi:10.3390/v11030204)
Supplement: Supplementary file 1 [file viruses-11-00204-s001.zip › TableS1.pdf]

Supplemental Table 1. List of human, canine, and feline noroviruses used for analyses.

Strain	Capsid Genotype	Host	Accession Number	Published Sequence	Reference
AN843	GIV.2	dog	MK067289	FL	This study
AN1610	GIV.2	dog	MK067288	FL	This study
AN1638	GIV.2	dog	MK067290	FL	This study
AN1663	GIV.2	dog	MK067291	FL	This study
AN1632	GVI.1	dog	MK067292	FL	This study
AN1633	GVI.1	dog	MK067293	FL	This study
AN1634	GVI.1	dog	MK067294	FL	This study
AN1640	GVI.2	dog	MK067295	FL	This study
AN1655	GVI.2	dog	MK067297	SG	This study
AN1656	GVI.2	dog	MK067296	SG	This study
170	GIV.2	dog	EU224456	Partial pol, SG	Martella et al., 2008
Thessaloniki 30	GIV.2	dog	GU354246	Partial pol, Partial VP1	Ntafis et al., 2010
Bari 91	GVI.1	dog	FJ875027	Partial pol, SG	Martella et al., 2008; 2009
FD210	GVI.1	dog	JF939046	Partial pol, SG	Martella, unpublished
5010	GVI.1	dog	KY486328	Partial pol, SG	Bodnar et al., 2017
63.15	GVI.2	dog	KY486329	Partial pol, SG	Bodnar et al., 2017
FD53	GVI.2	dog	JF930689	Partial pol, SG	Martella, unpublished
C33 Viseu	GVI.2	dog	GQ443611	Partial pol, SG	Mesquita et al., 2010
Pistoia 387	GIV.2	lion	EF450827	Partial pol, SG	Martella et al., 2007
CU081210E	GIV.2	cat	JF781268	FL	Pinto et al., 2012
M81	GIV.2	cat	LC389583	Nearly FL	Takano and Hohdatsu, unpublished
M49-1	GVI.1	cat	LC011951	Nearly complete ORF1	Takano et al., 2015
M49-1	GVI.1	cat	LC011950	VP1	Takano and Hohdatsu, unpublished
TE 77-13	GVI.2	cat	KT245136	Partial pol, SG	Di Profio et al., unpublished
Lake Macquarie_NSW2680	GIV.1	human	JQ613567	FL	Eden et al., 2012
CCDC_GR1113-59	GIV.1	human	KC894731	FL	Ao et al., 2014
Saint Cloud 624	GIV.1	human	AF414427	Partial pol, SG	Fankhauser et al., unpublished
Fort Lauderdale 560	GIV.1	human	AF414426	Partial pol, SG	Fankhauser et al., 2002
Alphatron 98-2	GIV.1	human	AF195847	VP1	Vinje and Koopmans, 2000
OC01017023	GIV.1	human	LC101824	VP1	Seto et al., unpublished
Canet-en-Roussillon H7477	GIV.1	human	KT030674	VP1	de Rougemont et al., unpublished
Ahrenshoop246	GIV.1	human	JQ970479	Partial pol, VP1, Partial VP2	Hoehne, unpublished
Rome 283	GIV.1	human	HG004598	Partial pol, Partial VP1	Muscillo et al., 2013
Rome 673	GIV.1	human	HG004592	Partial pol, Partial VP1	Muscillo et al., 2013
Rome 131	GIV.1	human	HG004588	Partial pol, Partial VP1	Muscillo et al., 2013
Rome 675	GIV.1	human	HG004602	Partial pol, Partial VP1	Muscillo et al., 2013
Rome 1	GIV.1	human	HG004603	Partial pol, Partial VP1	Muscillo et al., 2013
Rome 303	GIV.1	human	HG004604	Partial pol, Partial VP1	Muscillo et al., 2013
Rome 301	GIV.1	human	HG004605	Partial pol, Partial VP1	Muscillo et al., 2013
Rome 282	GIV.1	human	HG004606	Partial pol, Partial VP1	Muscillo et al., 2013
Rome 222	GIV.1	human	HG004607	Partial pol, Partial VP1	Muscillo et al., 2013
Rome 267	GIV.1	human	HG004608	Partial pol, Partial VP1	Muscillo et al., 2013
WI7002	GIV.3	human	KX907728	FL	Collins et al., 2013
Chiba 040502	unassigned	human	AJ844470	SG	Okada et al., 2005
Yuzawa Gira2HS	unassigned	human	KJ196291	FL	Park et al., unpublished
