# Supplementary material for: Genomics Analyses of GIV and GVI Noroviruses Reveal the Distinct Clustering of Human and Animal Viruses
Source: Viruses. 2019 Mar 1;11(3):204. doi: 10.3390/v11030204 (PMC6466045; doi:10.3390/v11030204)
Supplement: Supplementary file 1 [file viruses-11-00204-s001.zip › TableS2.pdf]

Supplemental Table 2. Amino acid differences within VP1 that differentiate the genotype of GIV noroviruses.

GIV VP1: Genotype-Specific AA Differences																				
	226	286	292	294	296	297	300	326	359	367	370	377	387	401	404	417	437	444	480	520
Human/GIV.1/Saint Cloud 624	P	Y	G	S	S	A	E	V	K	A	L	A	T	T	N	E	E	Q	E	N
Human/GIV.1/Fort Lauderdale 560	.	.	.	.	.	.	.	.	.	.	.	.	.	.	.	.	.	.	.	.
Human/GIV.1/Alphatron 98-2	.	.	.	.	.	.	.	.	.	.	.	.	.	.	.	.	.	.	.	.
Human/GIV.1/OC01017023	.	.	.	.	.	.	.	.	.	.	.	.	.	.	.	.	.	.	.	.
Human/GIV.1/Canet-en-Roussillon H7477	.	.	.	.	.	.	.	.	.	.	.	.	.	.	.	.	.	.	.	.
Human/GIV.1/CCDC GR1113-59	.	.	.	.	.	.	.	.	.	.	.	.	.	.	.	.	.	.	.	.
Human/GIV.1/Ahrenshoop246	.	.	.	.	.	.	.	.	.	.	.	.	.	.	.	.	.	.	.	.
Human/GIV.1/LakeMacquarie NSW268O	.	.	.	.	.	.	.	.	.	.	.	.	.	.	.	.	.	.	.	.
Human/GIV.3/WI7002	L	F	N	R	A	T	T	T	R	S	Q	M	V	I	R	K	V	A	A	S
Cat/GIV.2/CU081210E	S	L	S	A	V	R	P	A	T	I	R	L	G	V	G	Q	N	P	A	G
Lion/GIV.2/Pistoia 387	S	L	S	A	V	R	P	A	T	I	R	L	G	V	G	Q	N	P	A	G
Dog/GIV.2/170	S	L	S	A	V	R	P	A	T	I	R	L	G	V	G	Q	N	P	S	G
Dog/GIV.2/AN843	S	L	S	A	V	R	P	A	T	I	R	L	G	V	G	Q	N	P	S	G
Dog/GIV.2/AN1610	S	L	S	A	V	R	P	A	T	I	R	L	G	V	G	Q	N	P	S	G
Dog/GIV.2/AN1638	S	L	S	A	V	R	P	A	T	I	R	L	G	V	G	Q	N	P	S	G
Dog/GIV.2/AN1663	S	L	S	A	V	R	P	A	T	I	R	L	G	V	G	Q	N	P	S	G
