# Supplementary material for: Genomics Analyses of GIV and GVI Noroviruses Reveal the Distinct Clustering of Human and Animal Viruses
Source: Viruses. 2019 Mar 1;11(3):204. doi: 10.3390/v11030204 (PMC6466045; doi:10.3390/v11030204)
Supplement: Supplementary file 1 [file viruses-11-00204-s001.zip › TableS3.pdf]

Supplemental Table 3. Amino acid alignment within VP1 (aa 301-337) of GIV and GVI noroviruses.

		301	302	303	304	305	306	307	308	309	310	311	312	313	314	315	316	317	318	319	320	321	322	323	324	325	326	327	328	329	330	331	332	333	334	335	336	337
GIV.2	Cat/GIV.2/USA/2010/CU081210E	A	P	A	L	T	E	E	M	P	-	-	-	-	P	L	E	D	E	V	A	D	G	A	A	A	T	Y	T	L	A	R	A	A	D	T	T	L
	Cat/GIV.2/JPN/2017/M81	.	.	.	.	.	.	.	.	.	-	-	-	-	.	.	.	.	.	.	.	.	.	.	.	.	.	.	.	.	.	.	.	.	.	.	.	.
	Lion/GIV.2/ITA/2006/Pistoia 387	.	.	.	.	.	.	.	.	.	-	-	-	-	.	.	.	.	.	.	.	.	.	.	.	.	.	.	.	.	T	.	.	.	.	.	A	.
	Dog/GIV.2/ITA/2007/170	.	.	.	.	S	.	.	.	.	-	-	-	-	.	.	.	.	.	.	.	H	.	.	.	.	.	.	A	V	G	.	.	.	.	.	.	.
	Dog/GIV.2/USA/2011/AN843	.	.	.	P	S	.	.	.	.	-	-	-	-	.	.	.	.	.	.	.	.	.	V	.	D	.	.	A	I	G	.	.	.	.	.	.	.
	Dog/GIV.2/USA/2017/AN1610	.	.	.	P	S	.	.	.	.	-	-	-	-	.	.	.	.	.	.	.	.	.	V	.	D	.	.	A	I	G	.	.	.	.	.	.	.
	Dog/GIV.2/USA/2017/AN1638	.	.	.	P	S	.	.	.	.	-	-	-	-	.	.	.	.	.	.	.	.	.	V	.	D	.	.	A	I	G	.	.	.	.	.	I	.
	Dog/GIV.2/USA/2017/AN1663	.	.	.	P	S	.	.	.	.	-	-	-	-	.	.	.	.	.	.	.	.	.	V	.	D	.	.	A	I	G	.	.	.	.	.	I	.
GVI.1	Cat/GVI.1/JPN/2012/M49-1	C	E	G	E	D	D	A	L	D	-	-	Q	A	S	.	G	A	.	P	I	R	D	Q	.	P	G	L	R	T	Q	A	R	-	S	.	E	R
	Dog/GVI.1/ITA/2007/Bari 91	C	E	G	E	E	S	.	V	N	-	-	T	T	.	T	D	S	G	A	T	G	D	Q	T	P	E	V	R	T	G	T	R	-	A	.	Q	R
	Dog/GVI.1/ITA/2007/FD210	C	E	N	E	E	S	A	T	D	-	-	P	M	.	T	.	T	-	D	T	.	N	Q	P	P	E	V	R	A	Q	A	R	-	A	.	Q	R
	Dog/GVI.1/ITA/2009/5010	-	-	-	-	D	A	.	G	D	-	-	G	S	.	Q	.	A	S	D	G	E	D	E	C	Q	S	A	A	D	M	T	R	-	A	.	Q	R
	Dog/GVI.1/USA/2017/AN1632	C	E	G	E	E	G	.	I	S	-	-	T	T	.	T	.	N	G	.	T	N	D	Q	T	P	E	V	R	A	Q	P	R	-	A	.	Q	R
	Dog/GVI.1/USA/2017/AN1633	C	E	G	E	E	G	.	I	S	-	-	T	T	.	T	.	N	G	.	T	N	D	Q	T	P	E	V	R	A	Q	P	R	-	A	.	Q	R
	Dog/GVI.1/USA/2017/AN1634	C	E	G	E	E	G	.	I	S	-	-	T	T	.	T	.	N	G	.	T	N	D	Q	T	P	E	V	R	A	Q	P	R	-	A	.	Q	R
GVI.2	Cat/GVI.2/ITA/2013/TE 77-13	G	L	G	W	D	-	-	-	-	-	-	-	-	-	-	L	.	A	T	T	L	V	D	G	P	E	A	R	V	Q	A	R	I	S	V	S	A
	Dog/GVI.2/PRT/2007/C33 Viseu	G	V	D	H	.	D	Q	G	.	S	L	G	A	G	P	S	N	D	-	-	P	V	S	V	P	E	A	R	S	Q	T	R	T	S	V	S	A
	Dog/GVI.2/ITA/2007/FD53	G	V	D	H	.	D	Q	G	.	S	L	G	T	G	P	S	.	D	-	-	S	V	S	V	P	E	A	R	A	Q	T	R	T	G	V	S	A
	Dog/GVI.2/ITA/2015/63.15	G	V	D	H	.	D	Q	G	L	P	L	G	T	G	P	S	.	D	-	-	S	T	S	.	P	E	V	R	A	Q	A	R	T	S	V	S	A
	Dog/GVI.2/USA/2017/AN1640	G	V	D	H	.	D	H	N	S	P	P	R	A	E	S	L	.	T	T	I	P	T	D	G	P	E	T	R	A	Q	A	R	T	S	A	S	A
	Dog/GVI.2/USA/2017/AN1655	G	V	D	H	.	D	H	N	.	P	P	Q	A	E	P	L	.	T	T	I	P	T	D	G	P	E	T	R	A	Q	A	R	T	S	A	S	A
	Dog/GVI.2/USA/2017/AN1656	G	V	D	H	.	D	H	N	.	P	P	Q	A	E	P	L	.	T	T	I	P	T	D	G	P	E	T	R	A	Q	A	R	T	S	A	S	A
GIV.1	Human/GIV.1/USA/1998/Saint Cloud 624	.	S	T	E	A	D	.	R	-	-	-	-	-	-	-	-	-	-	-	-	-	-	-	-	-	-	-	-	-	-	A	V	G	S	F	D	V
	Human/GIV.1/USA/1998/Fort Lauderdale 560	.	S	S	E	A	D	.	R	-	-	-	-	-	-	-	-	-	-	-	-	-	-	-	-	-	-	-	-	-	-	A	V	G	S	F	D	V
	Human/GIV.1/NET/1998/Alphatron 98-2	.	S	P	E	A	D	.	R	-	-	-	-	-	-	-	-	-	-	-	-	-	-	-	-	-	-	-	-	-	-	A	V	G	S	F	D	V
	Human/GIV.1/JPN/2001/OC01017023	.	S	P	E	A	D	.	R	-	-	-	-	-	-	-	-	-	-	-	-	-	-	-	-	-	-	-	-	-	-	A	V	G	S	F	D	V
	Human/GIV.1/FRA/2011/Canet-en-Roussillon H7477	.	S	S	E	A	D	.	R	-	-	-	-	-	-	-	-	-	-	-	-	-	-	-	-	-	-	-	-	-	-	A	V	G	S	F	D	V
	Human/GIV.1/CHN/2011/CCDC GR1113-59	.	S	P	E	A	D	.	R	-	-	-	-	-	-	-	-	-	-	-	-	-	-	-	-	-	-	-	-	-	-	A	V	G	S	F	D	V
	Human/GIV.1/DEU/2012/Ahrenshoop246	.	S	S	E	A	D	.	R	-	-	-	-	-	-	-	-	-	-	-	-	-	-	-	-	-	-	-	-	-	-	A	V	G	S	F	D	V
	Human/GIV.1/AUS/2010/LakeMacquarie NSW268O	.	S	S	E	A	D	.	R	-	-	-	-	-	-	-	-	-	-	-	-	-	-	-	-	-	-	-	-	-	-	A	V	G	S	F	D	V
GIV.3	Human/GIV.3/USA/2016/WI7002	S	T	T	E	.	-	-	-	-	-	-	-	-	-	-	-	-	-	-	-	-	-	-	-	-	-	-	-	-	-	.	T	G	.	.	D	.
