# Supplementary material for: Genomics Analyses of GIV and GVI Noroviruses Reveal the Distinct Clustering of Human and Animal Viruses
Source: Viruses. 2019 Mar 1;11(3):204. doi: 10.3390/v11030204 (PMC6466045; doi:10.3390/v11030204)
Supplement: Supplementary file 1 [file viruses-11-00204-s001.zip › TableS4.pdf]

Supplemental Table 4. Number of amino acid differences (% differences) of the complete polymerase (total of 511 residues) within and between species.

	No.	Human	Canine	Feline
Human	3	28 (5.5%)		
Canine	8	116.3 (22.8%)	9.9 (1.9%)	
Feline	2	115.3 (22.6%)	20.4 (4.0%)	11 (2.2%)
